# Image Entropy for the Identification of Chimera States of Spatiotemporal Divergence in Complex Coupled Maps of Matrices

**DOI:** 10.3390/e21050523

**Published:** 2019-05-24

**Authors:** Rasa Smidtaite, Guangqing Lu, Minvydas Ragulskis

**Affiliations:** 1 Center for Nonlinear Systems, Kaunas University of Technology, Studentu 50-146, LT-51368 Kaunas, Lithuania; 2 Department of Applied Mathematics, Kaunas University of Technology, Studentu 50-318, LT-51368 Kaunas, Lithuania; 3 School of Electrical and Information Engineering, Jinan University, 206 Qianshan Road, Zhuhai 519070, China

**Keywords:** chimera states, coupled map lattice, nilpotent matrix

## Abstract

Complex networks of coupled maps of matrices (NCMM) are investigated in this paper. It is shown that a NCMM can evolve into two different steady states—the quiet state or the state of divergence. It appears that chimera states of spatiotemporal divergence do exist in the regions around the boundary lines separating these two steady states. It is demonstrated that digital image entropy can be used as an effective measure for the visualization of these regions of chimera states in different networks (regular, feed-forward, random, and small-world NCMM).

## 1. Introduction

Chimera state is a dynamical spatiotemporal behavior when structured patterns of coherence and incoherence occur. This phenomenon was first observed in a network of non-locally coupled identical oscillators [1]. The existence of chimera states has been investigated in theory [2,3,4] as well as it has been proved in several experiments [5,6,7].

Chimeras are observed in optical [7,8], chemical [9,10], neuronal systems [11,12]. Experimental verification of chimeras in the system of non-locally coupled Belousov-Zhabotinsky chemical oscillators in a two-dimensional array is reported in [10]. The relativistic quantum chimera state is uncovered in two-dimensional Dirac material systems where the manifestations of both integrable and chaotic dynamics may be controlled electrically [8]. The coexistence of coherent and incoherent states, known as chimeras, is particularly important for neuronal systems. These states have also been linked to Parkinson’s disease, epileptic seizures, and even to schizophrenia [11]. The occurrence of chimera states in two-dimensional and three-dimensional networks of Hindmarsh-Rose oscillators representing realistic models of neuronal ensembles is identified in [12].

Initially it was thought that chimeras can be observed only in networks of non-locally coupled oscillators [1]. Later studies revealed that besides non-locally connected networks [3,4,13,14,15,16], these states can be found in local [17,18,19] as well as in global [6,20] coupling topologies. Chimera patterns are analyzed in networks of Logistic maps with hierarchical connectivities [21]. The robustness of chimera patterns to inhomogeneities in a lattice of identical FitzHugh-Nagumo oscillators with irregular coupling topologies is demonstrated in [22]. Besides these symmetric coupling topologies, chimera states are also observed in Erdős-Rényi [23], small-world [24], scale-free [25], heterogeneous [26] networks. The emergence of chimeras in a multiplex network with two non-identical interconnected layers is investigated in [27]. It is shown that the range of parameters displaying chimera states in the first homogeneous layer is affected by the changes in coupling of the same nodes in the second layer. Neural modular network is analyzed in [28] where neurons are assumed to be connected with electrical synapses within their communities and with chemical synapses across them—these two coupling types cause the formation of chimera-like states. To evaluate behavior of neurons measures of synchronization, metastability, and chimera-like states are estimated. The study of multiscale network [29] observes how the appearance of chimera states in global ring is influenced by the changes in topology of subnetworks.

The current study is focused on the dynamics of complex coupled maps of matrices. It is demonstrated that chimera states of spatiotemporal divergence do exist in the regions around the boundary lines separating the quiet state and the diverged state. That highlights the importance of this paper (chimera states have not been previously explored in coupled maps of matrices). Moreover, chimera states of spatiotemporal divergence are investigated in different types of networks, including random networks. The exploration of the effects induced by the network structure and the development of entropy-based visualization technique for chimera states of spatiotemporal divergence are the main objectives of this paper.

## 2. Preliminary Notes and the Objective

### 2.1. A Network of Coupled Maps

A paradigmatic model of a lattice of translational invariance with periodic boundary conditions, comprising *m* real-valued, single-variable time-discrete maps that are coupled to their closest neighbors reads [14]:

(1) $$x^{\left(t+1\right)}\left(i\right)=f\left(x^{\left(t\right)}\left(i\right),a\right)+\frac{\varepsilon }{2P}\sum_{j=i-P}^{i+P}\left(f\left(x^{\left(t\right)}\left(j\right),a\right)-f\left(x^{\left(t\right)}\left(i\right),a\right)\right)$$

where *i* is the number of the node (i=1,2,…,m); *t* is discrete time (t=0,1,2,…); $x^{\left(t\right)}\left(i\right)$ is the scalar nodal variable; ε is the coupling parameter within the interval (0,1); *P* is a fixed number of nearest neighbors to either side (P≥0). The local dynamics of every element *i* on the one-dimensional ring is described by the Logistic map:

(2) $$f\left(x^{\left(t\right)}\left(i\right),a\right)=ax^{\left(t\right)}\left(i\right)\left(1-x^{\left(t\right)}\left(i\right)\right)$$

where 0<a≤4 and the initial condition is bounded to $0\leq x^{\left(0\right)}\left(i\right)\leq 1$ in order to ensure the mapping to the interval $x^{\left(t\right)}\left(i\right)\in \left[0,\;1\right]$ [30]. Please note that all parameters *a* of the Logistic map are identical for all nodes, but initial conditions $x^{\left(0\right)}\left(i\right)$ are randomly distributed in interval [0,1].

At P=1 Equation (1) describes a standard coupled map lattice (CML). At P≥2 Equation (1) represents a regular network of coupled maps. The coupling radius *r* is defined as $r=\frac{P}{m}$. Please note that r=0.5 ($r=\frac{m-1}{2m}$ if *m* is odd) corresponds to global coupling.

### 2.2. A Network of Coupled Map of Matrices

CMLs play an important role in modelling such complex phenomena as, spatiotemporal chaos, spatial bifurcations, global travelling waves [31,32,33]. A scalar iterative nodal variable at each node of a CML can be replaced by a matrix variable [34]. All scalar variables $x^{\left(t\right)}\left(i\right)$ are replaced by 2×2 matrices $\left[\begin{array}{cc} x_{11}^{\left(t\right)}\left(i\right) & x_{12}^{\left(t\right)}\left(i\right)\\ x_{21}^{\left(t\right)}\left(i\right) & x_{22}^{\left(t\right)}\left(i\right) \end{array}\right]$ in Equations (1) and (2). Such a transition from a scalar Logistic map (Equation (2)) to a single Logistic map of matrices is explained in detail in [35]. All square 2×2 matrices can be classified into idempotent and nilpotent matrices; however only nilpotent matrices can generate the effect of divergence in an isolated Logistic map of matrices when the absolute values of the matrix elements grow unbounded [35]. Therefore, all 2×2 matrices in this paper will be set as nilpotent matrices.

Please note that a 2×2 nilpotent matrix can be uniquely characterized by its single Eigenvalue $\lambda^{\left(t\right)}$ and a scalar nilpotent parameter $\mu^{\left(t\right)}$ [35]. Appropriate re-arrangements and the collection of terms do transform the CML described by Equation (1) and Equation (2) into a one-dimensional coupled map lattice of matrices (1D CMLM) [34]:

(3) $$\begin{array}{ccc} \lambda^{\left(t+1\right)}\left(i\right) & = & a\lambda^{\left(t\right)}\left(i\right)\left(1-\lambda^{\left(t\right)}\left(i\right)\right), \end{array}$$

(4) $$\begin{array}{ccc} \mu^{\left(t+1\right)}\left(i\right) & = & \left(1-\varepsilon \right)a\mu^{\left(t\right)}\left(i\right)\left(1-2\lambda^{\left(t\right)}\left(i\right)\right)+\frac{\varepsilon }{2}\left(a\mu^{\left(t\right)}\left(i+1\right)\left(1-2\lambda^{\left(t\right)}\left(i+1\right)\right)\right.\\ & & \;\left.+a\mu^{\left(t\right)}\left(i-1\right)\left(1-2\lambda^{\left(t\right)}\left(i-1\right)\right)\right), \end{array}$$

where $0\leq \lambda^{\left(0\right)}\left(i\right)\leq 1$ is the single Eigenvalue of the initial nilpotent matrix at node *i*; $\mu^{\left(0\right)}\left(i\right)=1$ (i=1,2,…,m) is the nilpotent parameter of the initial nilpotent matrix at node *i*. The nilpotent model of a 1D CMLM comprises two scalar maps—therefore the lattice parameters $\lambda^{\left(t\right)}\left(i\right)$ and $\mu^{\left(t\right)}\left(i\right)$ are computed directly instead of performing matrix computations on the 1D lattice [34]. Please note that the divergence of a node *i* is represented by the unbounded growth of $\mu^{\left(t\right)}\left(i\right)$.

The main objective of this paper is to investigate the dynamics of a network of coupled maps where scalar map variables are replaced by matrix variables. The model of such networks of coupled maps of matrices (NCMM) follows from Equations (1) and (3):

(5) $$\mu^{\left(t+1\right)}\left(i\right)=f\left(\mu^{\left(t\right)}\left(i\right),\lambda^{\left(t\right)}\left(i\right),a\right)+\frac{\varepsilon }{2P}\sum_{j=i-P}^{i+P}\left(f\left(\mu^{\left(t\right)}\left(j\right),\lambda^{\left(t\right)}\left(j\right),a\right)-f\left(\mu^{\left(t\right)}\left(i\right),\lambda^{\left(t\right)}\left(i\right),a\right)\right),$$

where

(6) $$f\left(\mu^{\left(t\right)}\left(i\right),\lambda^{\left(t\right)}\left(i\right),a\right)=a\mu^{\left(t\right)}\left(i\right)\left(1-2\lambda^{\left(t\right)}\left(i\right)\right)$$

but Eigenvalues of nilpotent matrices are computed directly according to Equation (3). At P=1 the NCMM reduces to a 1D CMLM which (as shown in [34]) can generate fractal patterns of $\mu^{\left(t\right)}\left(i\right)$ representing spatiotemporal divergence that can be controlled by the coupling parameter between the nodes.

In other words, the main objective of this paper is to investigate if NCMMs (at P≥2) can exhibit chimera states of spatiotemporal divergence. Such NCMMs will be called regular NCMMs due to the orderly connectivity of neighboring nodes.

## 3. Chimera States of Spatiotemporal Divergence in Regular NCMMs

### 3.1. Spatiotemporal Divergence in a Regular NCMM

**Figure 1:**
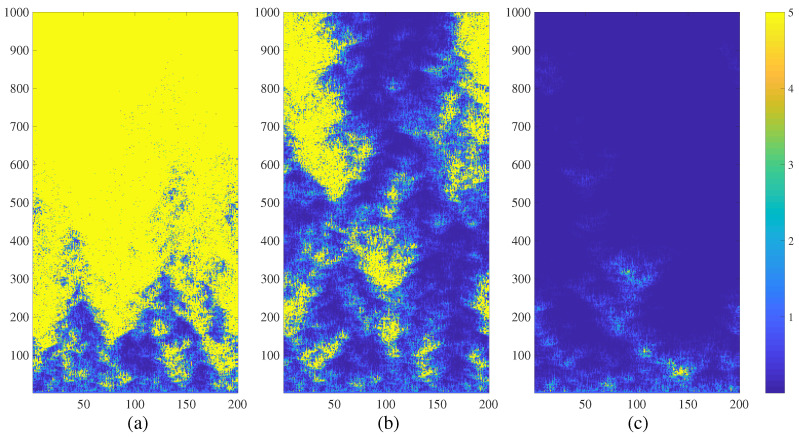
The transient dynamics of a regular NCMM comprising 200 nodes (a=3.699956; ε=0.4; $\lambda^{\left(0\right)}\left(i\right)$; i=1,2,…,200 are randomly distributed in the interval $\left(0,\;1\right)$) represented by the variation of $\mu^{\left(t\right)}\left(i\right)$. The network diverges at r=0.02 (part (**a**)); generates complex patterns at r=0.025 (part (**b**)); and calms down at r=0.03 (part (**c**)). Numerical values of $\mu^{\left(t\right)}\left(i\right)$ are truncated to 5 for the clarity of presentation.

A regular NCMM comprising 200 nodes is investigated in this section. The parameter of the Logistic map *a* is set to 3.699956 (the onset of chaos); the coupling parameter ε is set to 0.4. Initial Eigenvalues $\lambda^{\left(0\right)}\left(i\right)$; i=1,2,…,200 are randomly distributed in the interval $\left(0,\;1\right)$. The regular NCMM is iterated in 1000 time-forward steps according to Equation (5). The evolution of the network at P=4$\left(r=0.02\right)$; P=5$\left(r=0.025\right)$ and P=6$\left(r=0.03\right)$ is depicted in Figure 1 parts (a), (b) and (c) respectively.

The regular NCMM diverges after a turbulent transient process at P=4 (absolute numerical values of $\mu^{\left(t\right)}\left(i\right)$ are truncated to 5 in Figure 1a for the clarity of presentation). However, the regular NCMM calms down (Figure 1c) when each node is connected to 12 adjacent neighbors (P=6). It appears that the degree of connectivity can be used to control the divergence of the network.

It is interesting to observe that the evolution of the regular NCMM results into a complex pattern at P=5 (Figure 1b). The nodes are grouped into clusters of temporary divergence, however they calm down and re-explode again during the turbulent evolution of the network in time (Figure 1b). As mentioned in the Introduction, chimera states describe a dynamical spatiotemporal behavior when structured patterns of coherence and incoherence occur [1]. The definition of chimera states is extended in this paper. Figure 1b depicts a spatiotemporal behavior when structured patterns of quiet states and diverging states occur. The quiet state of a node *i* is defined as the state when $\mu^{\left(t\right)}\left(i\right)$ tends to zero. The spatiotemporal divergence of the node *i* is defined as the state when the modulus of $\mu^{\left(t\right)}\left(i\right)$ exceeds a pre-set level (this level is set to 5 in all computational experiments in this paper). Such behavior of the network is described as chimera states of spatiotemporal divergence. Such a complex behavior of the regular NCMM raises a question about the global view of the dynamics of the network in the parameter plane ε−r.

As mentioned previously, the standard definition of chimera states is modified to the definition of chimera states of spatiotemporal divergence in this paper. In other words, structured patterns of coherence and incoherence are replaced by structured patterns of quiet and diverging states. It would be tempting to rename the diverging states as chaotic states. Also, it must be noted that spatiotemporal chaos is a well-explored phenomenon in cellular automata [36].

**Figure 2:**
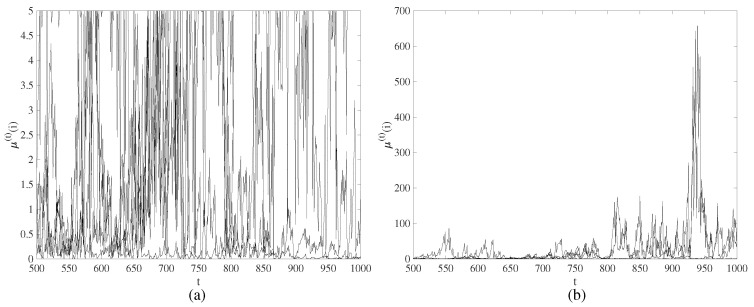
The evolution of $\mu^{\left(t\right)}\left(1\right)$, $\mu^{\left(t\right)}\left(50\right)$, $\mu^{\left(t\right)}\left(100\right)$, $\mu^{\left(t\right)}\left(150\right)$ and $\mu^{\left(t\right)}\left(200\right)$ in time interval 500≤t≤1000 at the set of system parameters corresponding to Figure 1b. The numerical values of $\mu^{\left(t\right)}\left(i\right)$ are cropped to 5 in part (**a**) and are shown uncropped in part (**b**).

However, transitional states of temporary divergence cannot be defined as chaotic transients. By the definition, a chaotic attractor is bounded in the phase space. In our model, the evolution of a nodal variable $\mu^{\left(t\right)}\left(i\right)$ is not bounded. This is illustrated in Figure 2 where the evolution of 5 nodes ($\mu^{\left(t\right)}\left(1\right)$, $\mu^{\left(t\right)}\left(50\right)$, $\mu^{\left(t\right)}\left(100\right)$, $\mu^{\left(t\right)}\left(150\right)$ and $\mu^{\left(t\right)}\left(200\right)$) is visualized in time interval 500≤t≤1000 at the set of system parameters corresponding to Figure 1b. The numerical values of $\mu^{\left(t\right)}\left(i\right)$ are cropped to 5 in Figure 2a—but the uncropped values of $\mu^{\left(t\right)}\left(i\right)$ are depicted in Figure 2b. It is clear from Figure 2 that the evolution of $\mu^{\left(t\right)}\left(i\right)$ cannot be described as the bursting chaos [37] (bounded in the phase space). Therefore, the definition of chimera states of spatiotemporal divergence is used in this paper.

Coherent states are represented as quiet states. However, diverging nodes evolve in radically different trajectories (Figure 2b)—what corresponds to the incoherent states.

The visualization of the transient dynamics of the NCMM at every point of the parameter plane ε−r poses serious technical problems. Instead, the regular NCMM is evolved until the transient processes cease down and the steady-state evolution of the network is registered for 150 time-forward steps. That results into a grayscale digital image representing the values of $\mu^{\left(t\right)}\left(i\right)$; 1≤i≤200; $0\leq \mu^{\left(t\right)}\left(i\right)\leq 5$ (the size of the digital image in pixels is 200×150). Then, this digital grayscale image representing the steady-state evolution of the network is reduced into one single scalar number representing the entropy of that image (we use the standard MATLAB function *entropy*).

**Figure 3:**
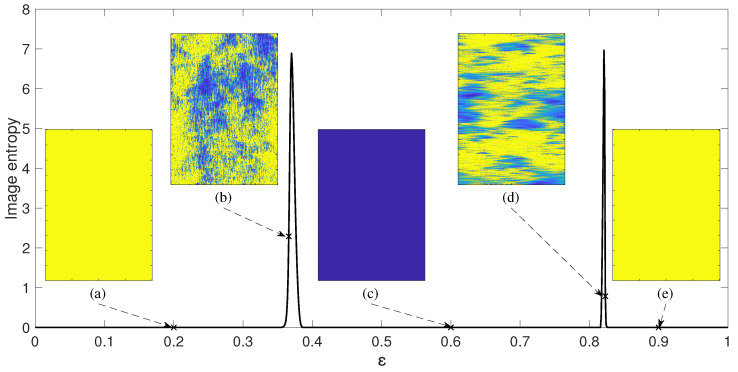
Image entropy of patterns is calculated for regular network when parameter *r* is set to 0.05. Network coupling parameter ε is set to 0.2, 0.366, 0.6, 0.823 and 0.9 in parts (**a**), (**b**), (**c**), (**d**) and (**e**). Image entropy is equal to 2.29 and 0.785 in parts (**b**) and (**d**) respectively.

The schematic diagram representing this information reduction process is illustrated in Figure 3. The parameter *r* is set to 0.05; all other system parameters (except ε) are kept the same. The coupling parameter ε is varied from 0 to 1 and image entropy is computed for the steady-state evolution of the regular NCMM for each discrete value of ε (Figure 3). Please note that the image entropy for the quiet network (Figure 3c) and the diverged network (Figure 3a,e) are all equal to zero. However, chimera-type states of spatiotemporal divergence yield entropies larger than zero (Figure 3b,d).

The relationship between the image entropy and the coupling parameter ε yields two distinct peaks in Figure 3. Such behavior of the regular NCMM is very interesting. Initially, when the coupling parameter ε is small, the network diverges (Figure 3). When the coupling parameter ε exceeds a critical value (over 0.38), the network’s final state is the quiet state (Figure 3). That corresponds well to the phenomenon observed in 1D CMLM—the effect of divergence can be controlled by increasing the coupling parameter ε [34]. However, a completely unexpected behavior of the regular NCMM is observed when the coupling parameter ε exceeds the upper threshold (around 0.83)—the network diverges again (Figure 3).

Such behavior of the regular NCMM reminds a coupled network of dendritic neurons [38]. A strongly coupled network of dendritic neurons tends to synchronize (what is dangerous to the functionality of brain). The well-known medical procedure known as “the gamma knife” can be used to eliminate synchronized tangles of dendritic neurons causing epileptic seizures. Simulation results in [38] show that the annihilation of too many synaptic links between neurons (caused by the overexposure of the network by a high dose of radiation therapy) leads to a synchronized state of the random network again. A similar effect can be observed in Figure 3—which shows an astonishing similarity (in terms of long-term behavior) between two networks of a completely different physical and mathematical origin.

Moreover, the regular NCMM exhibits a completely unique feature (compared to the network of dendritic neurons)—the dynamics of the network in the narrow region between the quiet mode and the divergence mode can be characterized by the existence of chimera states of spatiotemporal divergence (Figure 3b,d). Notably, image entropy detects the region of the existence of such chimera states very well (Figure 3).

**Figure 4:**
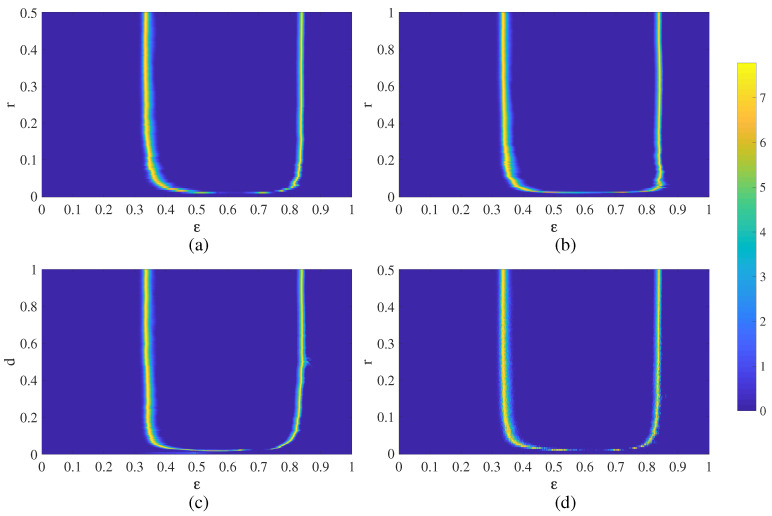
The visualization of chimera states of spatiotemporal divergence for networks of different structure: a regular NCMM (part (**a**), parameter plane ε−r); a regular unidirectional NCMM (part (**b**), parameter plane ε−r); the Erdős-Rényi NCMM (part (**c**), parameter plane ε−d); the small-world NCMM (part (**d**), parameter plane ε−r). The colorbar denotes numerical values of the entropy computed for steady-state evolution of the networks.

Finally, chimera states of spatiotemporal divergence can be identified in the whole parameter plane ε−r (Figure 4a). Chimera states of spatiotemporal divergence are located at the boundary between the quiet regime and the divergence regime (Figure 4a). The geometric shape of this boundary is very sensitive to the variation of *r* when *r* is small—but gets less sensitive when the regular network becomes denser (Figure 4a).

### 3.2. Chimera States of Spatiotemporal Divergence in a Regular Feed-Forward NCMM

Diffusive couplings between adjacent nodes is a paradigmatic choice for modelling neural networks which proves adequate in many cases [39]. However, feed-forward connectivity is also believed to play a significant role in a neuroscience context [40,41]. Each node is unidirectionally coupled to its successive neighbors in a feed-forward network:

(7) $$\mu^{\left(t+1\right)}\left(i\right)=f\left(\mu^{\left(t\right)}\left(i\right),\lambda^{\left(t\right)}\left(i\right),a\right)+\frac{\varepsilon }{P}\sum_{j=i}^{i+P}\left(f\left(\mu^{\left(t\right)}\left(j\right),\lambda^{\left(t\right)}\left(j\right),a\right)-f\left(\mu^{\left(t\right)}\left(i\right),\lambda^{\left(t\right)}\left(i\right),a\right)\right)$$

Please note that the coupling radius $r=\frac{P}{m}$ now ranges from $r=\frac{1}{m}$ for a local feed-forward network to $r=\frac{m-1}{m}$ for global unidirectional coupling.

**Figure 5:**
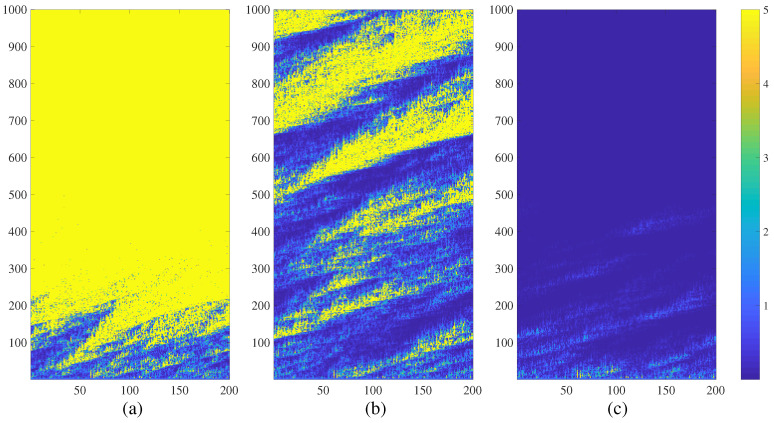
The transient dynamics of a regular directional NCMM comprising 200 nodes (a=3.699956; ε=0.4; $\lambda^{\left(0\right)}\left(i\right)$; i=1,2,…,200 are randomly distributed in the interval $\left(0,\;1\right)$) represented by the variation of $\mu^{\left(t\right)}\left(i\right)$. The network diverges at r=0.025 (part (**a**)); generates complex fractal-type patterns at r=0.035 (part (**b**)); and calms down at r=0.045 (part (**c**)). Numerical values of $\mu^{\left(t\right)}\left(i\right)$ are truncated to 5 for the clarity of presentation.

Computational experiments are continued with a regular feed-forward NCMM comprising 200 nodes (a=3.699956; ε=0.4; $\lambda^{\left(0\right)}\left(i\right)$ are randomly distributed in the interval $\left(0,\;1\right)$). The evolution of the network at P=5$\left(r=0.025\right)$; P=7 (r=0.035) and P=9 (r=0.045) is depicted in Figure 5 parts a, b, and c respectively.

The regular feed-forward NCMM diverges at P=5 (Figure 5a). The network exhibits chimera states of spatiotemporal divergence at P=7 (Figure 5b) and completely calms down at P=9 (Figure 5c). It is interesting to note that the feed-forward connectivity changes the shape of chimera states (Figure 5b)—the unidirectional coupling can be clearly identified from Figure 5.

The location of chimera states of spatiotemporal divergence for the regular feed-forward NCMM are shown in parameter plane ε−r in Figure 4b. Chimera states are located at the boundary between the quiet regime and the divergence regime—but a surprising is the fact that the geometric shape of this region is very similar to Figure 4a.

## 4. Chimera States of Spatiotemporal Divergence in a Complex NCMM

Most social, biological, and technological networks exhibit non-trivial topological features, with patterns of connection between their nodes that are neither purely regular nor purely random. Three relevant characteristics are usually employed to characterize a complex network—randomness, heterogeneity and modularity [42].

One extreme are regular networks. These are usually man-made networks that have the lowest heterogeneity and lowest randomness (as discussed in Section 3.1 and Section 3.2). Another extreme is random Erdős-Rényi networks [43]. Such random networks have low heterogeneity and the degree distribution will be a Gaussian bell-shaped curve. The emergence and visualization of chimera states of spatiotemporal divergence in a random Erdős-Rényi NCMM is investigated in Section 4.1.

Most real-world networks, however, do not have homogeneous distribution of degree that regular or random networks have. The number of connections each node has in most real-world networks varies greatly and they are positioned somewhere between regular and random networks. A typical real-world network is proposed in [44] where the connections between the nodes in a regular graph are rewired with a certain probability. The resulting networks can be positioned between the regular and random networks according to their topological structure—and are referred to as small-world networks. The emergence and visualization of chimera states of spatiotemporal divergence in a small-world NCMM is investigated in Section 4.2.

### 4.1. Chimera States of Spatiotemporal Divergence in the Erdős-Rényi NCMM

The Erdős-Rényi NCMM network is generated by starting with a disconnected set of nodes that are then paired with a uniform probability. The coupling density of the Erdős-Rényi NCMM is defined as the ratio between the existing number of edges $n_{r}$ and the maximum number of edges in a complete network: $d=\frac{2n_{r}}{m\left(m-1\right)}$. Please note that 0≤d≤1.

The model of the Erdős-Rényi network is adopted from [45]:

(8) $$\mu^{\left(t+1\right)}\left(i\right)=\left(1-\varepsilon \right)f\left(\mu^{\left(t\right)}\left(i\right),\lambda^{\left(t\right)}\left(i\right),a\right)+\frac{\varepsilon }{k_{i}}\sum_{j=1}^{m}T_{i,j}\left(d\right)f\left(\mu^{\left(t\right)}\left(j\right),\lambda^{\left(t\right)}\left(j\right),a\right),$$

where the mapping function *f* remains the same as in Equation (6); ε is the coupling parameter; ${}_{i}$ is the degree of the node *i*. The adjacency matrix $T_{i,j}$ represents the Erdős-Rényi random network where the average degree of node *i* is set to *d*. The iterative relationship for $\lambda^{\left(t\right)}\left(i\right)$ also remains the same as in Equation (3).

**Figure 6:**
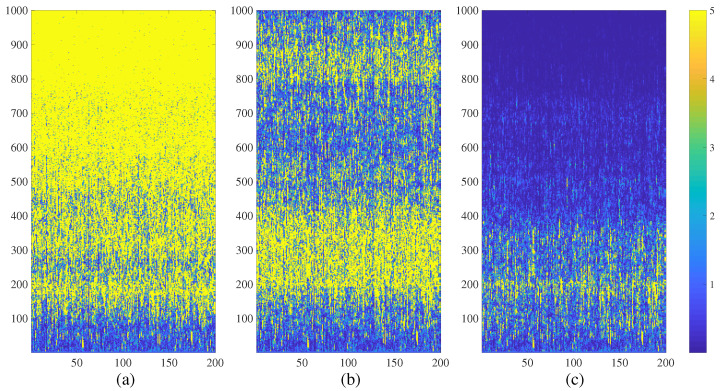
The transient dynamics of the Erdős-Rényi NCMM comprising 200 nodes (a=3.699956; ε=0.4; $\lambda^{\left(0\right)}\left(i\right)$; i=1,2,…,200 are randomly distributed in the interval $\left(0,\;1\right)$) represented by the variation of $\mu^{\left(t\right)}\left(i\right)$. The network diverges at d=0.031 (part (**a**)); generates complex fractal-type patterns at d=0.033 (part (**b**)); and calms down at d=0.035 (part (**c**)). Numerical values of $\mu^{\left(t\right)}\left(i\right)$ are truncated to 5 for the clarity of presentation.

The Erdős-Rényi NCMM diverges at d=0.031 (Figure 6a). The network exhibits complex transient states of spatiotemporal divergence at d=0.033 (Figure 6b) and completely calms down at d=0.035 (Figure 6c).

It is well-known that the visualization of chimera states in a random network poses serious technical problems because adjacent nodes do not necessarily belong to the same chimera state [25]. In other words, the visualization of interpretable chimera states requires special and not always clearly defined node permutation algorithms [25].

Despite the before-mentioned problems with the visualization of chimera states, we continue with the digital image entropy-based algorithm without the node permutation (Figure 4c). The results are surprising. First of all, the geometric shape of the region of chimera states of spatiotemporal divergence is very similar to Figure 4b. Secondly, the boundaries of the region of chimera states are smooth—the random nature of the network does not substantially change the geometric shape of the region.

### 4.2. Chimera States of Spatiotemporal Divergence in the Small-World NCMM

Computational experiments are continued with the small-world NCMM. To obtain a small-world network the Watts-Strogatz model is considered [44]. Watts-Strogatz network is constructed starting from a ring lattice with *m* nodes and *k* edges per node. Each pair of nodes is rewired with probability β. Please note that a regular network is generated at β=0. However, when all edges are rewired (β=1) a ring lattice is transformed into a random graph.

The implementation of the small-world network of CMM is similar to Equation (8) except that the adjacency matrix is computed according to the Watts-Strogatz model [44].

As a starting point a ring lattice with *P* nearest neighbors (Equation (1)) is considered—which results in the construction of undirected networks. The probability β to rewire the target node is set to 0.2 in all calculations.

**Figure 7:**
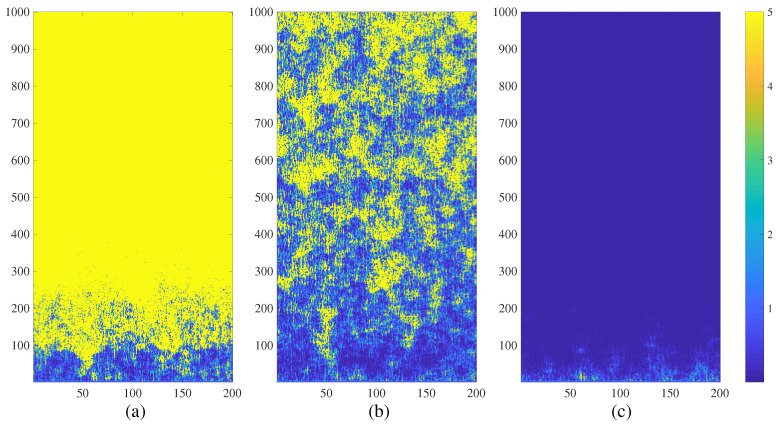
The transient dynamics of the small-world network NCMM comprising 200 nodes (a=3.699956; ε=0.4; $\lambda^{\left(0\right)}\left(i\right)$; i=1,2,…,200 are randomly distributed in the interval $\left(0,\;1\right)$; β=0.2) represented by the variation of $\mu^{\left(t\right)}\left(i\right)$. The network diverges at r=0.015 (part (**a**)); generates complex patterns of spatiotemporal divergence at r=0.02 (part (**b**)); and calms down at r=0.025 (part (**c**)). Numerical values of $\mu^{\left(t\right)}\left(i\right)$ are truncated to 5 for the clarity of presentation.

The Watts-Strogatz NCMM diverges at P=3 (r=0.015) in Figure 7 part (a). The network experiences transient processes of spatiotemporal divergence at P=4 (r=0.02) in Figure 7 part (b) and completely calms down at P=5 (r=0.025) in Figure 7 part (c).

Chimera states of spatiotemporal divergence for the small-world NCMM in the (r,ε) parameter plane are shown in Figure 4d. Surprisingly, the shape of the highlighted region is very similar to Figure 4a–c—even though the network topology is completely different.

## 5. Concluding Remarks

The visualization of chimera states in a regular one-dimensional lattice does not cause much difficulties because these chimera states are represented by compact time-varying clusters of synchronized nodes. However, the concept of the space is lost in complex networks, which makes it not straightforward to define a chimera state [25]. To enhance the view of chimera states, the rearrangement of nodes can be done. The node with the highest degree is labelled to be the first, then other nodes are arranged according to their distance from the first node [25].

The visualization scheme for chimera states in this manuscript is not based on the rearrangement on nodes. Moreover, chimera states in NCMM are not states of spatiotemporal synchronization between the neurons or other types of nonlinear oscillators. Chimera states in NCMM do exist in the regions around the boundary lines separating the quiet state or the state of divergence. These chimera states represent the self-organization of nodes into spatiotemporal clusters of divergence. It appears that image entropy is an effective measure for the visualization of the regions of chimera states in NCMM. Moreover, the proposed techniques work well with different topology networks (regular, feed-forward, random, and small-world NCMM). The network structure has a strong impact to the geometrical shape of chimera states of spatiotemporal divergence (compare Figure 1b, Figure 5b, Figure 6b, Figure 7b). However, it appears that the boundary line separating the quiet states and the diverged states is not strongly affected by the structure of the network—which is a completely counter-intuitive result. This robustness of the geometric shape of boundary lines against the network structure has important implications for different potential applications—desynchronization of complex coupled maps of matrices, temporary control of divergence in coupled maps of matrices, etc. These applications remain clear objectives of future research.

The existence (and appropriate visualization) of chimera states of spatiotemporal divergence is already an interesting result in nonlinear dynamics of complex CMLs of matrices. The sensitivity of these chimera states to different perturbations, the potential of chimera states to embed and to transmit secret visual information—these are important questions falling out of the scope of this paper—but remaining a definite objective of future research.
